# Resting-State Neurophysiological Abnormalities in Posttraumatic Stress Disorder: A Magnetoencephalography Study

**DOI:** 10.3389/fnhum.2017.00205

**Published:** 2017-04-25

**Authors:** Amy S. Badura-Brack, Elizabeth Heinrichs-Graham, Timothy J. McDermott, Katherine M. Becker, Tara J. Ryan, Maya M. Khanna, Tony W. Wilson

**Affiliations:** ^1^Department of Psychology, Creighton UniversityOmaha, NE, USA; ^2^Center for Magnetoencephalography (MEG), University of Nebraska Medical Center (UNMC)Omaha, NE, USA; ^3^Department of Neurological Sciences, University of Nebraska Medical Center (UNMC)Omaha, NE, USA; ^4^Department of Psychology, Colorado State UniversityFort Collins, CO, USA; ^5^Department of Psychology, Simon Fraser UniversityBurnaby, BC, Canada

**Keywords:** PTSD, MEG, resting-state, posttraumatic stress disorder, magnetoencephalography (MEG)

## Abstract

Posttraumatic stress disorder (PTSD) is a debilitating psychiatric condition that is common in veterans returning from combat operations. While the symptoms of PTSD have been extensively characterized, the neural mechanisms that underlie PTSD are only vaguely understood. In this study, we examined the neurophysiology of PTSD using magnetoencephalography (MEG) in a sample of veterans with and without PTSD. Our primary hypothesis was that veterans with PTSD would exhibit aberrant activity across multiple brain networks, especially those involving medial temporal and frontal regions. To this end, we examined a total of 51 USA combat veterans with a battery of clinical interviews and tests. Thirty-one of the combat veterans met diagnostic criteria for PTSD and the remaining 20 did not have PTSD. All participants then underwent high-density MEG during an eyes-closed resting-state task, and the resulting data were analyzed using a Bayesian image reconstruction method. Our results indicated that veterans with PTSD had significantly stronger neural activity in prefrontal, sensorimotor and temporal areas compared to those without PTSD. Veterans with PTSD also exhibited significantly stronger activity in the bilateral amygdalae, parahippocampal and hippocampal regions. Conversely, healthy veterans had stronger neural activity in the bilateral occipital cortices relative to veterans with PTSD. In conclusion, these data suggest that veterans with PTSD exhibit aberrant neural activation in multiple cortical areas, as well as medial temporal structures implicated in affective processing.

## Introduction

Posttraumatic stress disorder (PTSD) is a psychiatric disorder involving re-experiencing, avoidance, negative mood and cognition and arousal symptoms (American Psychiatric Association, 2013). The prevalence of PTSD in veterans of Operation Enduring Freedom (OEF) and Operation Iraqi Freedom (OIF) is estimated at 23% (Bagalman, 2013), which is about 2.5 times higher than the 8.9% USA lifetime prevalence rate of PTSD (American Psychiatric Association, 2013). Greater combat exposure is associated with more severe PTSD symptomatology, as well as greater psychiatric comorbidity in veterans (Dedert et al., 2009; Vasterling et al., 2010; Bagalman, 2013) consistent with the inter-relatedness of the core constructs of PTSD, anxiety and depression (Byllesby et al., 2016).

Previous fMRI and PET studies of PTSD have consistently reported aberrant activation in the anterior, middle and posterior cingulate cortices, medial prefrontal and middle frontal gyri, as well as the insula and medial temporal structures such as the hippocampus and amygdalae of patients with PTSD relative to matched controls (Rabinak et al., 2011; Morey et al., 2012; Pitman et al., 2012; Sripada et al., 2012; Boccia et al., 2016). Meta-analysis has suggested that the anterior cingulate cortices (ACC) and bilateral amygdala may be the most commonly hyper-activated regions in patients with PTSD (Hayes et al., 2012). Structural studies have also reported reduced volume in similar regions, including the hippocampal (O’Doherty et al., 2015) and ACC in patients with PTSD relative to controls (Meng et al., 2014; O’Doherty et al., 2015). Beyond the classic fronto-limbic regions, studies have reported abnormalities in multiple cortical areas including parietal regions, default-mode network areas, dorsal prefrontal regions, motor regions and occipital areas in those with PTSD compared to demographically-matched controls (Eckart et al., 2011; Schuff et al., 2011; Liu et al., 2012; Mueller-Pfeiffer et al., 2013). Thus, it seems unlikely that the disorder can be uniquely attributed to dysfunction in the fronto-limbic regions that have been historically implicated.

As with other areas of psychiatric and neurologic research, resting-state fMRI studies of PTSD have increased and several fMRI studies have reported altered resting-state functional connectivity in participants with PTSD compared to controls, especially in regions of the default-mode network (Qin et al., 2012; Zhou et al., 2012; Du et al., 2015; Reuveni et al., 2016). In addition, resting-state studies using magnetoencephalography (MEG) have appeared and these studies have suggested hyper-connectivity in medial temporal areas of patients with PTSD (Dunkley et al., 2014, 2015), as well as miscommunication between right temporo-parietal cortices and the rest of the brain (Engdahl et al., 2010). All of these studies focused on connectivity and those by Dunkley et al. (2014, 2015) used a mask that limited their analysis to specific *a priori* fronto-limbic areas. Thus, whether the regions themselves were dysfunctional (i.e., not just connectivity), and whether aberrant processing would have been detected beyond fronto-limbic regions in patients with PTSD remains unknown. One preliminary MEG study of traumatic memory recall in women with PTSD found activation largely outside of fronto-limbic regions (Cottraux et al., 2015), calling attention to other brain regions. In the current study, we examined a group of combat veterans with and without PTSD using resting-state MEG and clinical assessments. We focused on resting-state activity as it circumvents possible differences in behavioral performance from biasing the neural signals, and used MEG due to its excellent spatiotemporal precision and close relationship with the underlying neurophysiology. The two groups of veterans were closely-matched on important demographic variables, and following MEG their data were source imaged and evaluated using a voxel-by-voxel whole brain approach. Our primary goal was to identify brain regions with altered spontaneous neuronal activity in combat veterans with PTSD. Our central hypothesis was that veterans with PTSD would exhibit aberrant resting-state (i.e., spontaneous) activity in multiple cortical, medial temporal and fronto-limbic areas compared to veterans without PTSD.

## Materials and Methods

We recruited a community sample of 51 USA military veterans living near Omaha, NE, USA, who had experienced combat in conflicts in Iraq and Afghanistan (between 2003 and 2014) using television commercials, flyers, and social media. Following clinical assessment (see below), it was determined that 31 of these male combat veterans met the diagnostic criteria for PTSD and the remaining 20 male combat veterans did not meet criteria for PTSD or any other psychiatric or neurological condition. Participants without PTSD were matched to those with PTSD on age, education, ethnicity and handedness. General exclusionary criteria included any medical diagnosis affecting CNS function, known brain neoplasm or lesion, history of significant head injury and ferromagnetic implants. This study was carried out in accordance with the recommendations of the Creighton University Institutional Review Board with written informed consent from all subjects. All subjects gave written informed consent in accordance with the Declaration of Helsinki. The protocol was approved by the Creighton University Institutional Review Board.

### Psychological Assessment

PTSD was diagnosed using the Clinician Administered PTSD Scale (CAPS; Blake et al., 1995) and the symptom Frequency of 1, symptom Intensity of 2 rule (Weathers et al., 1999). Veterans in the healthy control group did not meet criteria for PTSD or any other psychiatric disorder. Participants were assessed for comorbid diagnoses using the Mini International Neuropsychiatric Interview (MINI; Sheehan et al., 1998), and none of the participants had a comorbid diagnosis of psychosis, bipolar disorder, obsessive-compulsive disorder, or current substance dependence. All participants also completed a battery of psychological questionnaires including the Patient Health Questionnaire (PHQ-9), which measured depression (Kroenke et al., 2001); the Toronto Alexithymia Scale (TAS-20), which measured difficulty identifying and describing feelings (Bagby et al., 1994); the State-Trait Anxiety Inventory (STAI), which measured both state and trait anxiety (Spielberger et al., 1983); the Deployment Risk and Resilience Inventory (DRRI), which measured combat exposure (Vogt et al., 2008); and the Life Events Checklist (LEC) measured traumatic events across the lifespan (Blake et al., 1995). Finally, as noted above, all veterans also completed the CAPS (Blake et al., 1995), which is the gold standard diagnostic interview for PTSD. The CAPS produced a PTSD symptom severity score for each participant (including those without PTSD), which we report in the results.

### MEG Data Acquisition and sMRI Coregistration

All recordings were conducted in a one-layer MSR with active shielding engaged. With an acquisition bandwidth of  0.1–330 Hz, neuromagnetic responses were sampled continuously at 1 kHz using an Elekta system with 306 magnetic sensors, including 204 planar gradiometers and 102 magnetometers (Elekta, Helsinki, Finland). MEG data from each participant were individually corrected for head motion and subjected to noise reduction using the signal space separation method with a temporal extension (tSSS; Taulu and Simola, 2006).

Prior to MEG measurement, four coils were attached to the participant’s head and the locations of these coils, together with the three fiducial points and scalp surface, were determined with a 3-D digitizer (Fastrak 3SF0002, Polhemus Navigator Sciences, Colchester, VT, USA). Once the participant was positioned for MEG recording, an electric current with a unique frequency label (e.g., 322 Hz) was fed to each of the coils. This induced a measurable magnetic field and allowed each coil to be localized in reference to the sensors throughout the recording session. Since coil locations were also known in head coordinates, all MEG measurements could be transformed into a common coordinate system. With this coordinate system (including the scalp surface points), each participant’s MEG data were coregistered with T1-weighted sMRI data using the Statistical Parametric Mapping software (SPM12; Litvak et al., 2011) implemented in Matlab (Mathworks, Inc.).

### MEG Preprocessing, Source Imaging and Statistics

The continuous 6-min magnetic time series was divided into epochs of 4096 ms duration, and epochs with artifacts were identified using an automated procedure that identified flat and high amplitude segments for rejection; this procedure was supplemented with visual inspection and ultimately resulted in less than 5% of epochs being rejected. Artifact-free epochs were filtered 1–54 Hz and then downsampled to 180 Hz prior to image reconstruction to reduce computational burden. The downsampled data across all 306 sensors were then imaged using the Bayesian multiple sparse priors (MSP) approach implemented in the SPM12 software (Litvak et al., 2011; Wellcome Trust Centre for Neuroimaging).

In our MSP approach, source space consisted of 8196 dipolar voxels equally-distributed throughout gray matter. Data covariance was calculated across the whole epoch from 1 Hz to 54 Hz. Prior to inversion, the MEG sensor data were transformed into a set of orthogonal modes using a singular value decomposition over the lead field matrix, and a temporal projector was applied to reduce the data to 16 temporal modes (Friston et al., 2008; Henson et al., 2009; López et al., 2014). A Variational Laplace approach was then used to estimate the combination of hyper-parameters that maximized free energy in the Bayesian framework. Model selection used both automatic relevance determination and greedy search schemes, and the covariance matrices produced by each and the sensor noise covariances were mixed using Variational Laplace in a second inversion step. The resulting single covariance matrix was used to get the posterior mean and variance of the current density, with output images written at 2 mm^3^ resolution. See López et al. (2014) for a detailed description of the MSP approach to Bayesian source reconstruction.

The resulting 3D maps of functional brain activity were statistically evaluated in SPM12 using a mass univariate approach based on the general linear model. Briefly, the effects of group were examined using a mixed random effects model, whereas one-sample *t*-tests were conducted to probe task effects in each group. Statistical maps for task effects were thresholded at *p* < 0.001; whereas group effect maps were thresholded at *p* < 0.01. All statistical maps were adjusted for multiple comparisons using a spatial extent threshold (cluster restriction), which was calculated directly from the data according to the theory of Gaussian random fields. All statistical analyses for behavioral and clinical variables were conducted in SPSS (Release 21.0.0).

## Results

### Participant Demographics and Clinical Measures

No participants were receiving psychotherapy at the time of enrollment. Eleven veterans with PTSD were on stable (no change for at least 6 months) doses of psychiatric medication (4 SSRIs, 3 benzodiazepines and 4 mood stabilizers), and the other 20 veterans with PTSD were not taking any psychiatric medication. One veteran in the non-PTSD group was taking a SSRI. Mean age was 32.13 (SD: 5.98) years in the PTSD group and 31.60 (SD: 7.18) years in the control group (*p* = 0.78). Mean educational level was 14.71 (SD: 2.41) years in the PTSD group and 14.13 (SD: 2.70) years in the control group (*p* = 0.42). By design, veterans with PTSD had significantly higher PTSD scores than the no PTSD comparison group (*p* < 0.0001). As expected, veterans with PTSD also reported higher rates of depressive and anxious symptoms compared to those without PTSD (*p*’s < 0.01; see Table 1). Such increased depressive and anxious symptoms are commonly associated with PTSD and are part of the typical clinical presentation of PTSD. Lastly, veterans with PTSD had significantly more severe combat exposure relative to those without PTSD (*p* < 0.05; Table 1) according to the DRRI, but the two groups did not statistically differ in exposure to lifelong trauma exposure including childhood and/or civilian trauma experiences.

**Table 1:** **Psychological assessment scores**.

Measure (symptom)	Group	Mean (SD)
CAPS (PTSD)**	PTSD	72.03 (15.31)
	Non-PTSD	22.95 (11.94)
PHQ-9 (depression)**	PTSD	11.77 (6.14)
	Non-PTSD	2.35 (2.28)
TAS-20 (alexithymia)**	PTSD	57.77 (13.57)
	Non-PTSD	42.40 (10.50)
STAI Y1 (state anxiety)**	PTSD	45.29 (9.85)
	Non-PTSD	35.90 (5.90)
STAI Y2 (trait anxiety)**	PTSD	47.65 (12.21)
	Non-PTSD	31.20 (6.94)
DRRI (combat exposure)*	PTSD	38.07 (17.21)
	Non-PTSD	26.59 (19.11)

PTSD (n = 31); Non-PTSD (n = 20). **Scores significantly different between groups at p < 0.01. *Scores significantly different between groups at p < 0.05.

### MEG Data: Resting-State Effects

**Figure 1:**
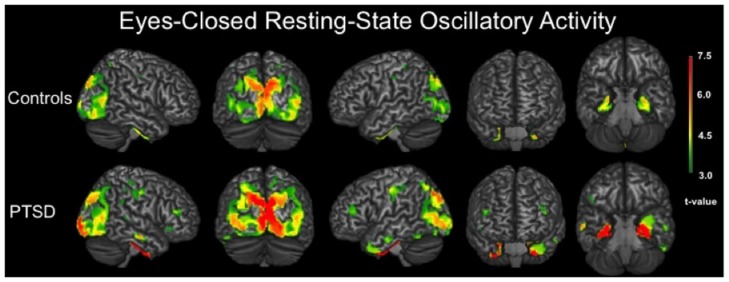
**3D renditions showing brain regions with significant resting-state neuronal activity in combat veterans without posttraumatic stress disorder (PTSD; top row) and with PTSD (bottom row)**. All group-level maps have been thresholded at (*p* < 0.001, corrected); a color scale bar showing respective *t*-values appears on the far right. Brain regions with significant activity in veterans without PTSD included bilateral occipital regions, inferior parietal cortices, postcentral gyri, posterior cingulate, parahippocampal gyri and other medial temporal areas. For veterans with PTSD, bilateral activity was observed in the primary motor and somatosensory cortices (i.e., precentral and postcentral gyri), superior parietal cortices, inferior parietal cortices, occipital regions, dorsolateral prefrontal cortices, inferior temporal sulci, posterior cingulate and bilateral medial temporal structures including parahippocampal gyri, hippocampi and the amygdala.

We evaluated the brain areas active in veterans with and without PTSD during the resting-state prior to examining group differences. In both groups, significant neuronal activity was observed in bilateral areas of the occipital, inferior parietal, posterior cingulate and sensorimotor cortices (*p* < 0.001, corrected). Significant activity was also detected in bilateral medial temporal structures, including amygdalae, hippocampi and parahippocampal regions of each group (*p* < 0.001, corrected). In veterans with PTSD, bilateral prefrontal activity was also found (*p* < 0.001, corrected; Figure 1).

### MEG Data: PTSD Group Comparisons

**Figure 2:**
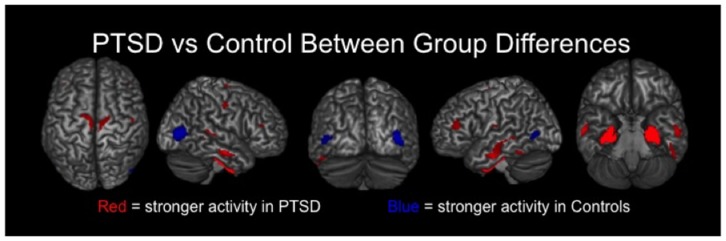
**Group differences in resting-state neuronal activity between combat veterans with and without PTSD were found in several brain regions**. In the top row, brain areas exhibiting significantly (*p* < 0.01, corrected) stronger activity in veterans with PTSD are shown in red, whereas regions with significantly weaker activity in these patients appear in blue. As shown, veterans with PTSD had stronger resting-state neuronal activity in bilateral medial temporal areas (e.g., amygdalae, parahippocampal), sensorimotor, temporal and prefrontal regions, along with significantly weaker activity in left and right lateral occipital cortices.

Veterans with PTSD exhibited significantly stronger neural activity in bilateral regions of the precentral and postcentral gyri, paracentral lobule, middle temporal gyri, inferior temporal sulci, DLPFC and the right superior temporal sulcus compared to veterans without PTSD (*p* < 0.01, corrected; Figure 2). Veterans with PTSD also had significantly stronger activity in the bilateral amygdala, parahippocampal and hippocampal regions relative to those without PTSD (*p* < 0.01, corrected). In contrast, veterans without PTSD had stronger activity in the left and right lateral occipital cortices compared to veterans with PTSD (*p* < 0.01, corrected; Figure 2).

In addition, we performed exploratory analyses to identify the primary frequency of neural activity underlying our critical findings, and as a sanity check to ensure high-frequency responses were not missed due to the broadband approach of our primary analysis (i.e., low-frequency responses are generally stronger than high-frequency and can be missed using such approaches). To this end, we filtered MEG signals into conventional frequency bands (e.g., theta, alpha) and imaged each band separately. Our results indicated that theta (4–7 Hz) was the critical frequency for our medial temporal findings (e.g., amygdala, parahippocampal, hippocampal), while alpha (8–14 Hz) activity was central to our occipital, temporal and sensorimotor findings. Our findings in the DLPFC extended across theta and alpha (i.e., group differences were observed in the DLPFC for both bands). Beta and gamma differences were much weaker, non-significant and likely made a negligible contribution to our primary findings.

## Discussion

We used high-density MEG and advanced image reconstruction methods to quantify resting-state (i.e., spontaneous) activity in combat veterans with and without PTSD. Our main findings were that veterans with PTSD exhibited significantly stronger activity relative those without PTSD in bilateral motor regions, lateral temporal areas, the DLPFC and multiple medial temporal areas including the hippocampi and amygdala. The opposite pattern of stronger activity in the non-PTSD group was observed in the lateral occipital cortices. Major strengths of this study included the thorough clinical assessments, state-of-the-art neurophysiological imaging, and the close matching of veterans with PTSD to a psychiatrically-healthy combat-exposed comparison group. Lifetime traumatic events were similar between groups, and although combat exposure was greater in the PTSD group, the comparison sample had also experienced significant combat-related traumas. Given that the experience of trauma in the absence of PTSD symptoms appears to have a lasting effect on the brain (Stark et al., 2015), being able to draw conclusions specifically about the presence of PTSD requires a trauma exposed control group, yet such a control group is often absent in PTSD brain research. Below, we discuss the implications of these findings for understanding the neural circuitry of combat-related PTSD.

In each group, we observed significant resting-state neural activity in bilateral occipital, inferior parietal and sensorimotor cortices, as well as medial temporal structures including bilateral amygdala, hippocampus and parahippocampal cortices. In fact, with the exception of the DLPFC (observed in PTSD group only), both groups exhibited very similar patterns of resting-state neural activity. Group comparisons of the patients with PTSD and matched combat-exposed controls revealed regionally-specific patterns of increased and decreased neuronal activity during the resting-state. Specifically, we found stronger spontaneous neural activity in the bilateral sensorimotor cortices (pre and postcentral gyri), paracentral lobule and DLPFC in veterans with PTSD as compared to non-PTSD controls. The sensorimotor and prefrontal findings are consistent with evidence of aberrant neurodynamics in veterans with PTSD in these same brain regions in two recent MEG task-based studies (Badura-Brack et al., 2015; McDermott et al., 2016), as well as the results of a group classification study (Gong et al., 2014). The latter study indicated that the primary motor and prefrontal cortices were among the most important brain areas for group prediction, along with parietal and occipital regions (Gong et al., 2014). As expected, our PTSD group also had significantly stronger resting-state activity in medial temporal areas known to be critical for threat alert and emotion, including the hippocampi, parahippocampal gyri and amygdalae (Eckart et al., 2011; Morey et al., 2012; Meng et al., 2014). Interestingly, we also observed significantly weaker activity in the left and right lateral occipital cortices in veterans with PTSD compared to those without PTSD, which is congruent with other findings such as reduced occipital gray matter in PTSD (Chao et al., 2012; Li et al., 2014), and the resting-state predictive value of the occipital lobes in PTSD (Gong et al., 2014).

The amygdala, hippocampus and surrounding structures are perhaps the most well-documented brain regions in PTSD (Rabinak et al., 2011; Morey et al., 2012; Pitman et al., 2012; Sripada et al., 2012), and these areas significantly differentiated veterans with and without PTSD in our study. These regions, along with the parahippocampal gyri, are critical memory structures along the ventral visual processing stream, and are central to how traumatic information is stored and processed in PTSD (Brewin et al., 1996, 2010). We also found significant and widespread differences in more dorsal visual processing areas, suggestive of dorsal stream aberrations in PTSD. Influential theories of PTSD symptomatology suggest that re-experiencing (a key symptom) events are derived from visual sensory impressions that were inadequately integrated into autobiographical memory at the time of the trauma (Brewin et al., 1996, 2010; Ehlers and Clark, 2000). Involuntary memory intrusions in PTSD tend to have visual details predominating (Ehlers et al., 2002; Hackmann et al., 2004), and posttraumatic symptoms may be related to priming of these visual memories. Both sensorimotor and occipital areas along the dorsal processing stream appear to differentiate posttraumatic flashbacks from other autobiographical memories (Whalley et al., 2013). Our findings clearly highlight the need for future research into these brain regions, which have been woefully under-examined in the pathophysiology of PTSD.

In conclusion, we examined the neural basis of PTSD in a sample of USA combat veterans using advanced MEG methods and clinical assessments. We focused on the amplitude of resting-state (i.e., spontaneous) neural activity using a voxel-based whole brain approach, as previous studies had largely restricted their analyses to *a priori* brain regions and focused exclusively on functional connectivity among regions included in the *a priori* mask. Our results indicated that veterans with PTSD had stronger resting-state neural activity in bilateral areas of the occipital, inferior parietal, sensorimotor and parahippocampal cortices, as well as the amygdalae and hippocampi. The opposite pattern was observed in lateral occipital cortices bilaterally. In summary, PTSD is associated with aberrations in widespread brain regions, including dorsal neocortical areas and the classic medial temporal structures often linked to affective processing and PTSD symptomatology. Future task-based studies should closely evaluate their data for baseline level differences (i.e., preceding stimulus onset), as our findings suggest that spontaneous neural activity may be altered in PTSD. Future studies should also evaluate women and PTSD resulting from different types of trauma to determine whether the neurophysiological effects differ from what we observed in male combat veterans.

Finally, our study sample was not without limitations, including our focus on men and combat-related PTSD, possible medication or anxiety effects in the veterans with PTSD, and expected differences in the severity of combat exposure between the two groups. In addition, we examined only the resting state using MEG (i.e., no comparison task), and some of our findings in the medial temporal lobe will need to be confirmed by future studies given the reduced sensitivity of MEG to neural sources distant from the sensors. That said, we used both magnetometers and gradiometers for source reconstruction, and magnetometers are inherently more sensitive to neural activity in distant brain regions. We also observed anticipated group differences in these medial temporal structures, providing converging evidence on the veracity of these findings, as MEG recordings would be equally sensitive (or insensitive) to such activity across both groups. MEG studies reporting neural activity in deeper brain structures such as the cerebellum, amygdala and other medial temporal regions are becoming more common across the field (Kessler et al., 2006; Dalal et al., 2008; Salvadore et al., 2009, 2010; Wilson et al., 2009, 2010, 2011, 2017; Cornwell et al., 2012a,b, 2014; Muthuraman et al., 2014; McDermott et al., 2016; Proskovec et al., 2016; Pu et al., 2017). In summary, our MEG results converge nicely with well-established findings in these deeper structures in PTSD, draw needed attention to multiple cortical regions including expected prefrontal structures, as well as calling for increased consideration of sensorimotor, temporal and occipital areas in the pathophysiology of PTSD.

## Author Contributions

All authors certify having participated sufficiently in the work to take public responsibility for the content, including participation in the concept, design, analysis, writing and/or revision of the manuscript.

## Funding

This research was supported by a grant from the nonprofit organization At Ease, USA (AB-B), grant R01-MH103220 from the National Institutes of Health (TWW), and grant #1539067 from the National Science Foundation (TWW). The funders had no role in study design, data collection and analysis, decision to publish, or preparation of the manuscript.

## Conflict of Interest Statement

The authors declare that the research was conducted in the absence of any commercial or financial relationships that could be construed as a potential conflict of interest.
